# Hepatocellular carcinoma in infancy and childhood in Ibadan, Western Nigeria.

**DOI:** 10.1038/bjc.1967.55

**Published:** 1967-09

**Authors:** A. O. Williams, G. M. Edington, P. C. Obakponovwe

## Abstract

**Images:**


### HEPATOCELLULAR CARCINOMA IN INFANCY AND CHILDHOOD IN IBADAN, WESTERN NIGERIA

A. OLUFEMI WILLIAMS, G. M. EDINGTON AND P. C. OBAKPONOVWE

*From the Department of Pathology, University of Ibadan, Ibadan, Nigeria*

Received for publication October 27, 1966

ALTHOUGH primary malignant tumours of the liver occur rarely in early life, there are quite a number of cases in the literature from different parts of the world. These cases have been reviewed from time to time and additional cases have been recorded (Steiner, 1938; Drummond and Tollman, 1939; Edmondson, 1956; Clatworthy and Boles, 1961; Kasai, 1963; Fish, McCarey and Galveston, 1966; Misugi *et al.*, 1966). Reviewing the literature, there are no reports of these tumours in several parts of Africa, particularly in those areas where the incidence of adult hepatomas is relatively high and pregnant women who are poorly nourished are known to drink or eat locally produced medicinal preparations. Some of these are infusions of plants which have been shown to contain hepatotoxic alkaloids or similar carcinogenic compounds (Mattocks *et al.*, 1961; Schoental, 1963; Ogan, 1966, personal communication). Malignant liver cell tumours do not appear to be very common in our autopsies of infants and children but they are not uncommon in young adults. This paper deals with four cases of primary malignant liver cell tumours in Nigerian children seen in the University College Hospital, Ibadan, over a period of three years, 1963–1966. Two were diagnosed on open surgical biopsy specimens and the remaining two were diagnosed at autopsy.

### CASE REPORTS

### *Case I*

The patient, a moderately well developed 4-month-old male baby, weighing 4365 g., was first seen at the outpatients department of the University College Hospital at the age of 6 weeks. The mother had noticed gradual swelling of the abdomen, which progressively had got bigger since the child was about one month old. The child was suspected of having " worms " and constipation at that time and was treated with antihelminthics. He showed little or no improvement and the parents gave him local medicinal infusions orally for 3 weeks which they claimed were of some value. He then developed a skin rash, and was brought back to hospital when he was treated symptomatically. Two months later the presenting complaints were dyspnoea, anorexia, constipation and his mother had noticed a lump moving in his abdomen while the child fed. There had been no other significant illnesses. Examination at this stage revealed a grossly emaciated, dehydrated, afebrile child with no clinical evidence of jaundice, peripheral oedema or superficial lymphadenopathy. The mucous membranes were extremely pale and the abdomen was swollen. The child was dyspnoeic and some accessory muscles of respiration were being used. Pulse was 142/minute, regular and of poor volume, heart sounds were normal. Auscultation of the lungs revealed presence of adventitious sounds consistent with bronchopneumonia. Peristaltic movements were noted in the abdominal cavity and bowel sounds were diminished and indistinct. There was an ill defined mass in the left side of the abdomen which was stony dull to percussion and moved with respiration. Investigations carried out included haemoglobin : 4·9 g./100 ml. (34%), W.B.C.: 4450 per c.mm. with a normal differential, P.C.V. 15%, M.C.H.C.: 33%.

The clinical impression was a low intestinal obstruction probably Hirschsprung's disease or intussusception. The child went downhill rapidly and died on the day following admission.

*Pathology*

Necropsy was performed 48 hours after death. The body was that of an emaciated, dehydrated infant with a uniformly distended abdomen. On opening the abdominal cavity, the left cupola of the diaphragm was displaced upwards by a large hepatic mass in the left lobe. The stomach was displaced to the right and compressed backwards, the transverse colon and loops of small intestines were pushed downwards into the pelvis and the spleen appeared compressed and displaced downwards. Both kidneys remained unaffected. There was no free fluid in the peritoneal cavity. The main findings were confined to the liver and lungs. The liver was grossly enlarged, weighing 610 g. The enlargement was due to a tumour mass measuring 10 × 6 cm. occupying the left lobe but encroaching on the right lobe of the liver (Fig. 1). The capsule overlying the tumour was thickened with fibrous tissue. On section, the tumour mass was circumscribed and separated from the rest of the liver by a fibrous capsule. It was subdivided into nodules of various sizes by fibrous connective tissue radiating from the capsule. The nodules were multicoloured, some greenish yellow and bile stained, some appeared reddish while others were white or grey. Areas of mucoid degeneration and haemorrhages were present. The extrahepatic bile ducts and portal vein were patent and there was no enlargement of the porta hepatis group of lymph nodes. The inferior vena cava was patent. Radiographs of the liver tumour at autopsy showed no areas of calcification. There were areas of bronchopneumonia in the lower lobes of both lungs and compression atelectasis of the lower lobe of the left lung was also present.

*Histology of liver:* The capsule of the liver was thickened with fibrous tissue, particularly overlying the tumour. There was no invasion of subcapsular lymphatics or the fibrous capsule by neoplastic cells. There were fibrous bands of varying thickness dividing masses of tumour cells into nodules. In some areas the tumour cells were relatively large, columnar or cuboidal in shape and had an abundance of eosinophilic cytoplasm while in others the tumour cells were rather small. The nuclei were central, vesicular in shape and some had prominent nucleoli. There was considerable variation in the size of the nuclei and mitotic figures were infrequent. The tumour cells were arranged in cords or around small vessels thus simulating the lobular architecture of the normal liver. In the tumour there was a striking absence of portal tracts or their constituents but small quantities of bile pigment were present in some liver cells. In some areas foci of coagulation and haemorrhagic necrosis of liver cells were present. Aggregates of haemopoietic cells were prominent in the tumour (Fig. 2) but not in the unaffected lobe of liver. Sections from the right lobe of the liver showed no neoplastic cellular invasion except in the vicinity of the capsule of the tumour. There was diffuse fatty change of the liver cells in the right lobe and a few cells showed lytic necrosis. Portal tracts showed no changes and there was no cirrhosis.

*Lungs:* showed bronchopneumonia and patchy atelectasis. All other organs examined showed no changes.

*Summary:* Primary hepatocellular carcinoma (left lobe) with extra-medullary haemopoiesis, atelectasis left lung and bronchopneumonia.

*Case II*

The patient (F.O.) a 3-year-old male infant, was first seen at the Seventh Day Adventist Hospital, Ife, Western Nigeria, with a history of abdominal swelling of rapid onset. There was no vomiting, diarrhoea or abdominal pain. Physical examination revealed an emaciated child with a palpable hepatomegaly. Laparotomy revealed a large, inoperable, whitish tumour from which a biopsy was taken. The impression at operation was lymphosarcoma for which he was referred to University College Hospital, Ibadan. Soon after admission on 29th December, 1962, the abdomen became noticeably bigger and the child became severely dyspnoeic and died on the 2nd of January, 1963. Unfortunately the biopsy specimen referred to this department was lost in transit but an autopsy was carried out 20 hours after death.

*Necropsy:* The body was that of a grossly emaciated child with a 5 cm. long recent surgical incision in the upper abdomen. The main findings were confined to the abdominal cavity. The whole liver was grossly enlarged (1825 g.) and was adherent to duodenum, hepatic flexure and part of transverse colon and head of pancreas. The external surface of the liver was irregularly nodular and the capsule was thickened in places. On section, almost the whole of the parenchyma was destroyed by several bile stained greenish tumour nodules which varied in sizes and shapes. Both lobes of the liver were evenly involved with tumour and the gall bladder was completely infiltrated by tumour. Tumour deposits were also seen in peripancreatic lymph nodes. The portal vein and extrahepatic bile ducts were patent and normal. The spleen was slightly enlarged (100 g.) and there were a few accessory spleens. The trigone of the bladder mucosa was slightly granular. All the other organs including heart, lungs, kindeys, adrenals, testes and brain were normal. Haemoglobin electrophoresis of post mortem blood was AA.

*Histology:* Sections from the liver revealed that there was a primary tumour

---

EXPLANATION OF PLATES.

FIG. 1.—Tumour mass in left lobe of liver.

FIG. 2.—Microscopic appearance of tumour with aggregates of haemopoietic cells. H. & E. × 250.

FIG. 3.—Abdominal swelling due to carcinoma of liver in 4½-year-old female child. White line indicates lower border of liver.

FIG. 4.—Scanty reticulin and abnormal sinusoidal pattern of liver tumour. Gordon & Sweet. × 330.

FIG. 5.—Abnormal rows and cords of well differentiated malignant liver cells. H. & E. × 170.

FIG. 6.—Tumour cells in a dilated blood sinus. H. & E. × 160.

FIG. 7.—Autopsy radiographic appearance of secondary neuroblastoma in liver simulating hepatocellular carcinoma.

FIG. 8.—Histological appearance of a secondary neuroblastoma in liver showing neuroblastic cells and a fibrillary stroma. H. & E. × 200.

1

2

Williams, Edington and Obakponovwe.

3

4

Williams, Edington and Obakponovwe.

5

6

Williams, Edington and Obakponovwe.

7

8

Williams, Edington and Obakponovwe.

of liver cells. The capsule of the liver was thickened with fibrous tissue or tumour cells in places. Several aggregates of malignant cells were studded throughout the parenchyma and surrounded by normal compressed liver cells. The tumour cells were small and uniform and had large vesicular nuclei and eosinophilic cytoplasm. They were arranged in a trabecular fashion and mitosis was infrequent. There was moderate centrizonal fatty infiltration of liver cells but there was no cirrhosis. There was slight intrahepatic cholestasis but the portal tracts were normal. Metastatic tumour cells, similar to those in the liver, were seen in organs adherent to the liver. There were no other significant changes in other organs examined.

*Summary:* Primary hepatocellular carcinoma, metastases to lymph nodes with tumour invading gall bladder, duodenum, colon and pancreas.

*Case III*

The patient (K.A.) was a 4½-year-old female child who was referred to this hospital from Seventh Day Adventist Hospital, Ife, Western Nigeria. She presented with a 2-month history of abdominal swelling which had been getting progressively bigger. She was anorexic and had been losing weight but there was no abdominal pain. There was no history of jaundice, vomiting or diarrhoea. On examination she was rather dull looking but cooperative. There was mild lymphadenopathy of the axillae and groins. Pulse was 116/min. regular, good volume. The heart was not clinically enlarged and both lungs were clear. There was a palpably enlarged mass in the upper right hypochondrium extending downwards for about 17·5 cm. below the costal margin and 19 cm. in width (Fig. 3). The mass was firm with well defined antero-lateral edges and there was moderate ascites. The mass was thought to be either hepatic or renal in origin. The spleen was also enlarged 5 cm. below the left costal margin, felt smooth, but firm. The clinical diagnosis was somewhat in doubt, but differential diagnoses of hepatocellular carcinoma, Burkitt's lymphoma or Wilm's tumour were considered. Investigations included the following: haemoglobin 8 g./100 ml. (55%), W.B.C. 9700 with a normal differential count. M.C.H.C. 35%, P.C.V. 23%. Haemoglobin genotype: AA, S.G.O.T.: 41 Cabaud units/ml. of serum, S.G.P.T.: 23 Cabaud units/ml. of serum. Thymol turbidity: 10·4 units, Thymol flocculation +++, Total bilirubin: 1·6 mg./100 ml., Alkaline phosphatase: 11 King Armstrong units/100 ml. Total proteins: 7·9 g./100 ml. Electrophoresis showed slight depression of albumin and marked increase of gamma globulins. Intravenous pyelography showed normal functioning kidneys. Urinalysis revealed presence of calcium oxalate crystals and a few leucocytes but no bile or excess urobilinogen. Examination of stools revealed a few hookworm ova and undigested globules of fat. Electrolytes were normal. Blood cultures were negative. Blood urea—25 mg./100 ml. Chest X-ray showed no abnormalities but a flat X-ray of the abdomen showed a large, soft tissue mass in the right side of the abdomen with foci of calcification, probably a large hepatic tumour mass. Laparotomy revealed enlargement of the right lobe of the liver due to a large, inoperable, vascular tumour. A biopsy of the tumour was taken; other organs examined at operation appeared normal. She was given cytotoxic drugs and followed up for 6 months when she defaulted and it was not possible to contact her.

*Histology:* Sections from the biopsy specimen showed it to be entirely composed of a primary new growth of liver cells. The capsule was thickened with fibrous tissue and was well vascularised. The tumour was made up of sheets or groups of cells resembling those of the hepatic parenchyma but they were smaller in size. Mitotic figures were infrequent even in poorly differentiated areas. The reticulin content of the tumour was scanty, the architectural outlines of the reticulin framework was abnormal (Fig. 4) and was of diagnostic value particularly in well differentiated areas of the tumour. Prominent features of the tumour were the presence of broad fibrous bands composed chiefly of wavy hyaline fibrous tissue and collagen and increased vascularity. Tumour cells were seen in blood vessels but there was no evidence of extrahepatic metastases. Occasional groups of lymphocytes and mononuclears were also seen in the sinusoids and portal tracts.

*Summary:* Hepatocellular carcinoma with foci of calcification.

*Case IV*

The patient (M.F.) was a 10-year-old male child who presented in 1964 with abdominal swelling which he himself said he could see moving, could feel and had been painful particularly after meals and on palpation. He took some native medicinal infusions for 2 weeks before his admission but this made no difference to his complaint. In fact, it produced " black stools ". His appetite was good and he had not lost weight. His bowels opened about three times a day and micturition was normal. On examination, he was a thin, intelligent boy with pale mucous membranes but not jaundiced. The cardiovascular system was normal. He had an epigastric mass extending below the costal margin into the hypochondrium, its inferior border being 13 cm. below the tip of the xiphisternum. It was irregular, firm and tender, moved with respiration and moderately dull to percussion. There was no clinical evidence of ascites. Investigations carried out included: haemoglobin 81%, Haemoglobin electrophoresis AS, and the sickling test was positive, W.B.C. were 8900 with an absolute lymphocytosis. Liver function tests were normal and examination of stools revealed the presence of ova of Ancylostoma, Ascaris and Trichuris. Laparotomy carried out revealed a very large inoperable tumour mass involving the right lobe of liver which was solid and highly vascular on the surface. A biopsy was taken for histological diagnosis.

*Histology.*—Sections from the biopsy revealed a primary malignant liver cell tumour. The capsule was grossly thickened with fibrous tissue. The tumour was vascular and was made up of small cubical cells which were identical with parenchymal liver cells and were arranged in sheets or in abnormal rows or cords (Fig. 5). In areas the tumour was poorly differentiated and was associated with a scanty, abnormal, reticulin framework. Tumour cells were present in large blood sinuses (Fig. 6). There were dense bands of loose fibrocellular tissue containing several blood vessels with endarteritis. The portal tracts contained their constituent arteries and veins with a slight increase of lymphocytes but there was a striking absence of bile ductules.

*Summary.*—Hepatocellular carcinoma with vascular invasion.

*Follow Up.*—He was discharged and given haematinics as supportive therapy and had been followed up regularly for about 2½ years at the Tumour Therapy Clinic. When seen recently the hepatic tumour mass was still present and had remained about the same size. He felt very well and had been putting on weight but on no occasion had he been given cytotoxic drugs.

### DISCUSSION

The terminology of liver tumours in early life is confusing. For this paper, the term hepatocellular carcinoma, used by Edmondson (1956), is adopted for primary malignant liver cell tumours instead of hepatoblastoma. There are advantages and disadvantages for using the alternative term which has recently been applied to tumours in children under the age of three (Misugi *et al.*, 1966). These workers suggested that primary malignant tumours of the liver in infancy and childhood can be divided into two groups on the basis of age incidence, ultrastructure and histological appearances. Hepatoma was used for malignant tumours in children over the age of three because their histological appearances simulated adult tumours. While the term " hepatoblastoma " indicates a liver tumour of primitive or embryonal origin, it is not invariable that these tumours occur in children under the age of three. In fact, the tumours in all our cases were composed almost entirely of embryonal liver cells on light microscopy but two out of the four patients were over the age of 4. Since the biological behaviour of liver cell carcinomas is the same irrespective of age of onset, it is probably not essential to apply two titles to the same tumour.

The aetiological factors associated with hepatocellular carcinoma are many and these may vary with age and environment (Hou, 1958). The extremely early age at which liver carcinomas have occurred is noteworthy. Wilbur, Wood and Willett (1944) reported a case at birth thus indicating that the onset of neoplasia occurred *in utero.* Bigelow and Wright in 1953 collected about 50 cases in infants under the age of two years and 35 cases under the age of one year. A few of these tumours in childhood have been reported to be associated with hepatic cirrhosis (Macreary, 1937; Hamburger, 1938; Balasingham and Sreenivasan, 1938; Clatworthy and Boles, 1961; Cleland, 1959; Alcaide, Traisman and Baffes, 1962; Bloch and Chazan, 1956) not unlike what obtains with tumours in adults. Of possible aetiological importance is the fact that women in Ibadan are known to drink alkaloid-containing herbal infusions during their pregnancies, but there is still a lack of studies to incriminate any local substance capable of damaging foetal liver *in utero.*

The mother of the child (Case I) admitted to taking several local medicinal preparations during her pregnancy but when interviewed regarding their nature and composition, refused to volunteer any information. She also administered other similar infusions to the child but this was after the onset of his illness. There is no available information regarding the medicinal drinking habits of other patients or their mothers.

Although hepatocellular carcinoma in adults accounts for about 8% of all malignancies registered in our cancer registry*, the tumour in infancy and childhood is infrequent particularly when compared with tumours of the reticulo-endothelial system in children of comparable age.

The criteria for diagnosis of malignancy in the present cases include vascular invasion, rapid growth of tumour, metastases or invasive propensities, dedifferentiation, abnormal arrangement of tumour cells and abnormal reticulin and sinusoidal pattern. The histological diagnosis of carcinoma of the liver in infancy is usually not difficult but it should be distinguished from secondary neuro-

* The Cancer Registry in the department is supported by the British Empire Cancer Campaign for Research.

blastoma in the liver. This can be achieved by examing several sections of the tumour and identifying rosettes of Homer Wright or neuroblastic cells. We have seen a case which simulated hepatic carcinoma clinically and histologically in a 4-month-old boy but on examining several sections from the tumour, it was evident that it was secondary neuroblastoma because of the presence of immature type of neuronal nuclei amidst a fibrillary stroma (Fig. 7, 8).

No definite clinical picture appears to accompany the development of these neoplasms. The location of the tumour and the mechanical effects of the mass seem, in part, to dictate the clinical pattern and account for the variability of signs and symptoms. Usually the first recognizable sign is enlargement of the abdomen with development of a palpable mass in the upper abdomen. Pain in the region of the mass is not uncommon; anorexia, loss of weight and anaemia occur frequently. Jaundice and ascites are not common but when they occur, they are due to mechanical obstruction of the biliary ducts or branches of the portal vein. Fever appears late in the course of the disease. The disease untreated runs a rapid and progressive course and the average duration of life from the appearance of symptoms is approximately about 4 to 5 months (Bigelow and Wright, 1953; Fish, McCary and Galveston, 1966). If the tumour is situated in the right lobe of the liver a mis-diagnosis of Wilm's tumour is usually made, but when it affects the left lobe of the liver it may remain quiescent for some time until it starts to grow rapidly and then it produces intestinal symptoms by exerting mechanical effects on the neighbouring viscera.

In none of our cases was jaundice or pyrexia a prominent feature. The ages of the patients were 4 months, 3 years, 4½ years and 10 years respectively with a sex ratio of 3 males to 1 female. The tumour was in the left lobe in one case, in the right lobe in two cases and in both lobes in another case. From a review of 130 cases reported in the literature between 1953 and 1965, there was no difference in the sex ratio. Analysis of 63 cases showed that 76% were in Caucasians, 11% in Negroes, 11% in Orientals and 2% in the Latin race (Fish, McCary and Galveston, 1966).

In Case I the liver was about four times bigger than normal for his age, weighing 610 g. instead of 160 g. (Coppoletta and Wolbach, 1933). The tumour was situated in the left lobe, and this with the fact that the patient presented with symptoms of low intestinal obstruction would account for late diagnosis of the mass. The striking histological feature was the presence of extramedullary haemopoietic cells confined almost entirely to the tumour but absent in the normal portions of the liver examined. Although the tumour appeared encapsulated, tumour cells were seen just outside the capsule invading normal liver tissue which was moderately infiltrated with fat. The rapid downhill course of the illness is in keeping with a malignant disease. In Case II, the tumour affected both lobes of liver but the right lobe was much more affected and the gall bladder and head of pancreas were involved in the tumour mass. The liver was at least 4 times bigger than normal weighing 1825 g. instead of 418 g. (Coppoletta and Wolbach, 1933). The gross distribution of the tumour was different from the other cases. There were several tumour nodules scattered throughout the liver. These may be intrahepatic metastases from a primary site. The possibility of focal nodular hyperplasia was excluded by histology and the presence of extrahepatic metastases. Case III, however, was the only female and the only patient given cytotoxic drugs. She was initially given Mitomycin C, but this was stopped because of alopecia and haematological complications, and later Methotrexate was administered. Although the child was followed up for 6 months after discharge from hospital, the size of the liver was only slightly reduced when last seen. Unfortunately it has proved impossible to determine what has happened to her since then. Case IV was the oldest of our patients and had the longest history of abdominal swelling. He also took native medicinal preparations but it is likely that these were ingested after the onset of the illness. The occurrence of melaena after the intake of the native medicine is probably fortuitous and its significance and aetiology remain obscure. However it is noteworthy that his haemoglobin genotype was AS and his erythrocytes were positive for the sickling test. The tumour affected the right lobe of the liver and was inoperable. He did not receive any cytotoxic drugs and has been followed up for about 2½ years. Clinically, the size of the hepatic mass remains about the same and the child still feels very well. The length of survival of this patient, though untreated, is longer than the others, but this is not unusual. There are few reports on the results of surgical excision, radiotherapy and chemotherapy of this tumour of childhood, but the available information indicates that the prognosis is invariably fatal following chemotherapy, indeterminate after radiation therapy and only fair following surgical excision. In fact, Kasai (1963) concluded that the incidence of operable cases of hepatocellular carcinoma was higher in children than adults; they tolerated resection of the liver better than adults, and resection should be attempted in children if there were no evidence of metastases.

### SUMMARY

Four cases of hepatocellular carcinoma in infancy and childhood in Nigeria are described. Although hepatocellular carcinoma is not an uncommon tumour in adult Nigerians, it is relatively rare in infancy and childhood. The adult hepatocellular carcinomas commonly coexist with cirrhosis but this is rare in childhood tumours. The aetiology of the condition in early life remains obscure but the probable relationship to some local herbal remedies which contain alkaloids is commented upon. The clinicopathological features and diagnosis of the cases are described and discussed.

We wish to thank Professors Ngu and Hendrickse for allowing us to publish their cases, and the staff of the Medical Illustration Unit for the photographs.

### REFERENCES

Alcaide, M., Traisman, H. S. and Baffes, T.—(1962) *Am. J. Dis. Child.*, **104**, 245.
Balasingham, T. and Sreenivasan, B. R.—(1938) *J. Malaya Brch Br. med. Ass.*, **2**, 98.
Bigelow, N. H. and Wright, A. W.—(1953) *Cancer, N.Y.*, **6**, 170.
Bloch, H. and Chazan, S.—(1956) *Archs Pediat.*, **75**, 89.
Clatworthy, H. W. and Boles, E. T.—(1961) *Ann. Surg.*, **154**, 475.
Cleland, R. S.—(1959) *Pediat. Clins N. Am.*, **6**, 427.
Coppoletta, J. M. and Wolbach, S. B.—(1933) *Am. J. Path.*, **9**, 55.
Drummond, D. H. and Tollman, J. P.—(1939) *Am. J. clin. Path.*, **9**, 361.
Edmondson, H. A.—(1956) *Am. J. Dis. Child.*, **91**, 168.
Fish, J. C., McCary, R. G. and Galveston, T.—(1966) *Archs Surg.*, **93**, 355.
Hamburger, H. J. A.—(1938) *Indian J. Pediat.*, **5**, 98.

Hou, P. C.—(1958) ' Cancer ', Vol. 2, Edited by R. W. Raven. London (Butterworths & Co. Ltd.), p. 168.
Kasai, M.—(1963) *Surgery, St. Louis*, **54**, 351.
Macreary, T. W.—(1937) *Penn. med. J.*, **40**, 630.
Mattocks, A. R., Schoental, R., Crowley, H. C. and Culvenor, C. C. J.—(1961) *J. chem. Soc.*, 5400.
Misugi, K., Hiroyuki, O., Nobuko, M. and Newfon, W. A. Jr.—(1966) *Am. J. Path.*, **48**, 53a.
Schoental, R.—(1963) *Aust. J. Chem.*, **16**, 233.
Steiner, M. M.—(1938) *Am. J. Dis. Child.*, **55**, 807.
Wilbur, D. L., Wood, D. A. and Willett, F. M.—(1944) *Ann. intern. Med.*, **20**, 453.


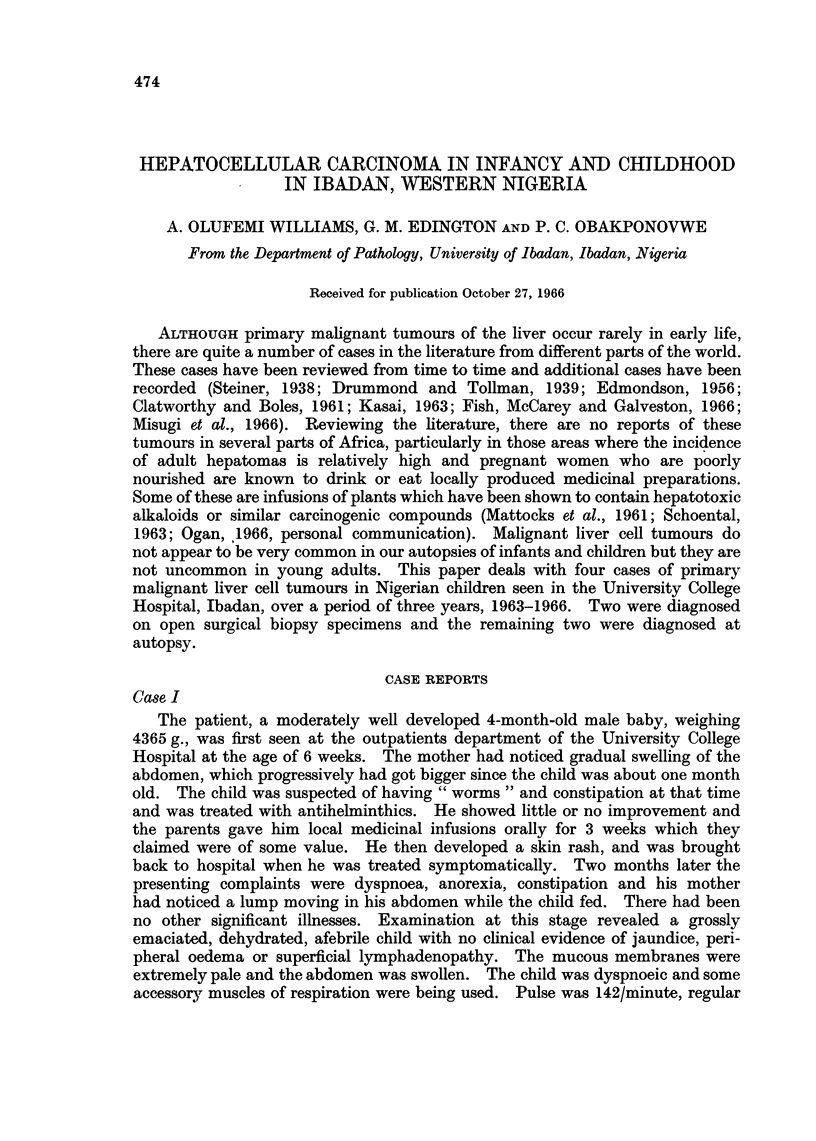

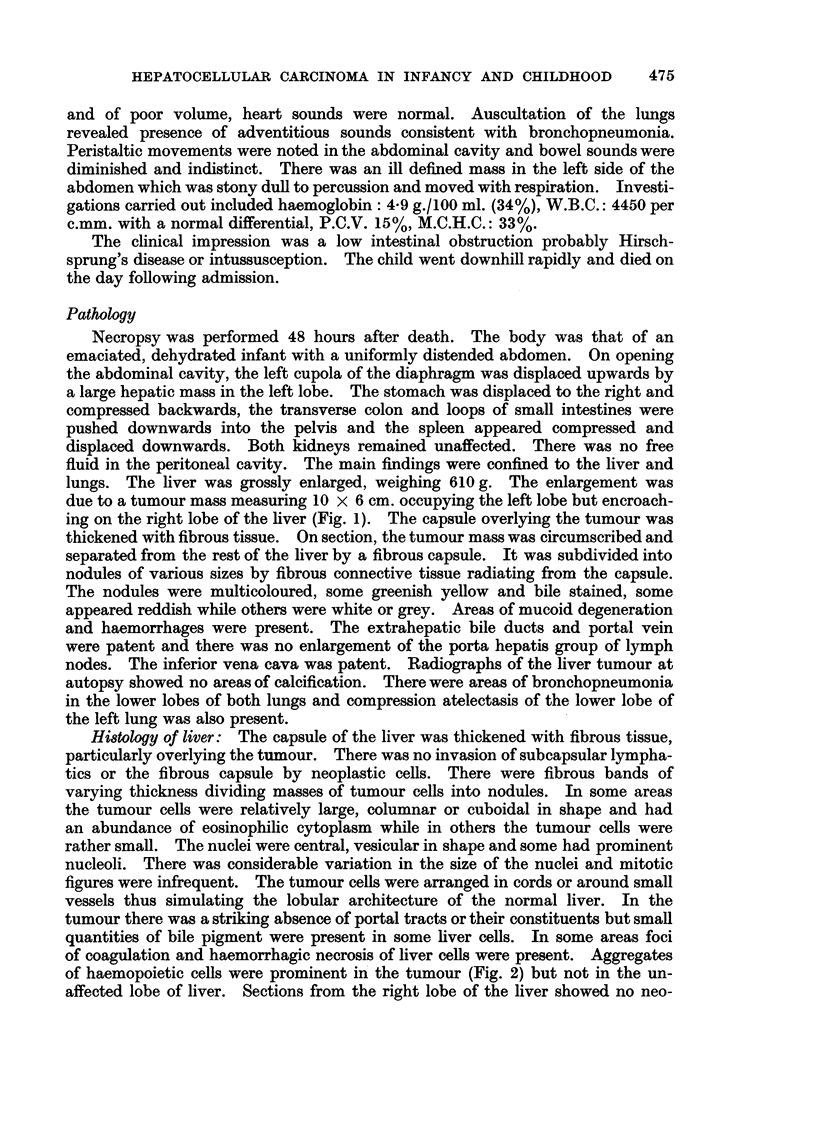

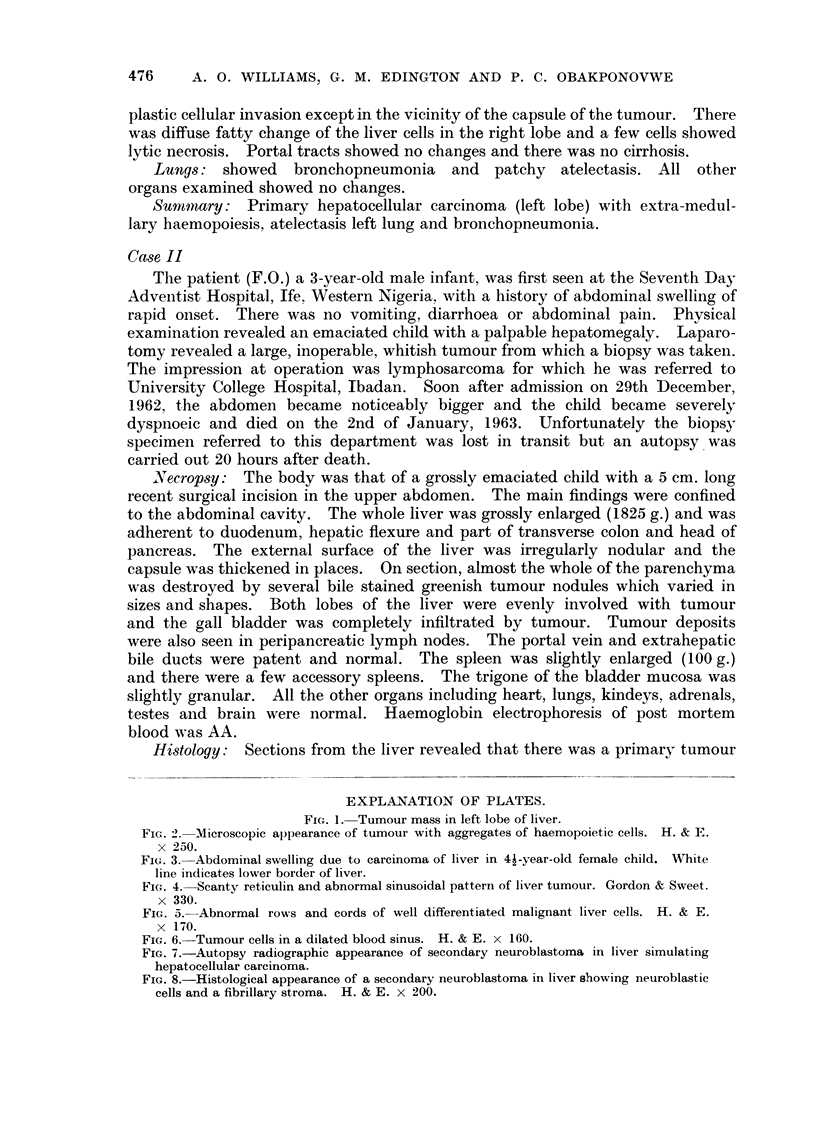

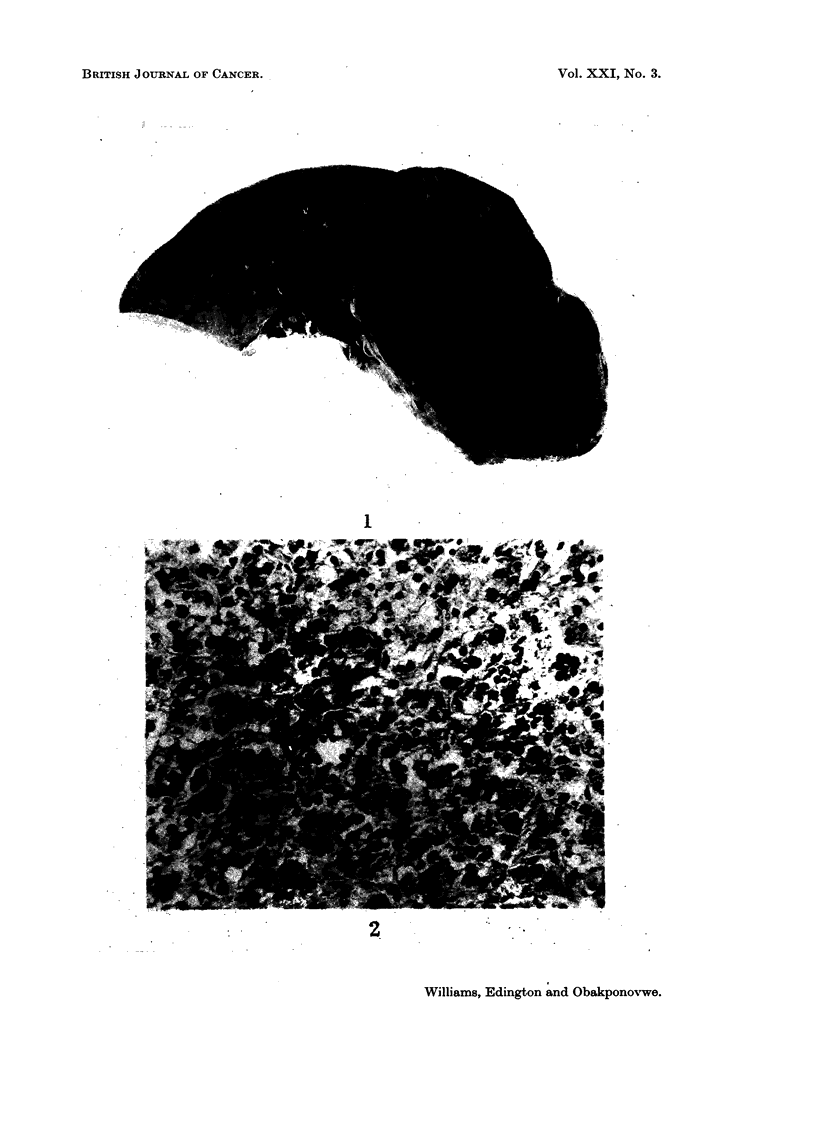

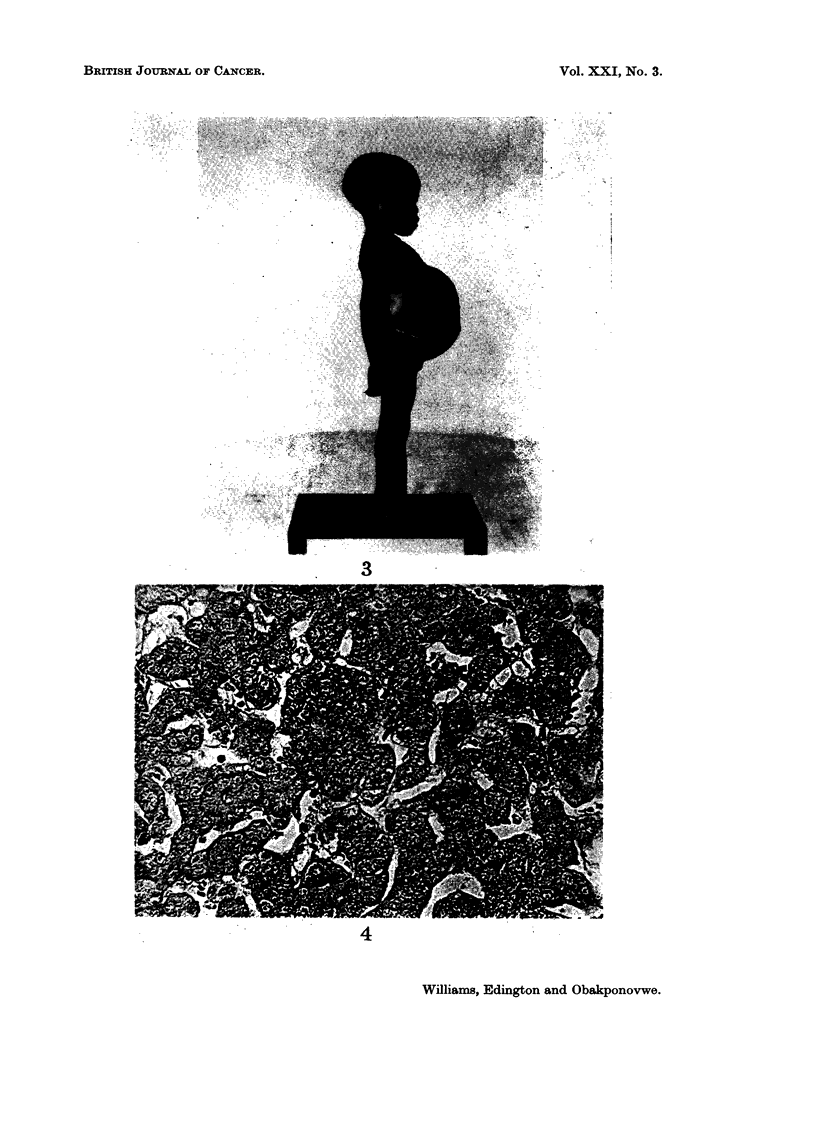

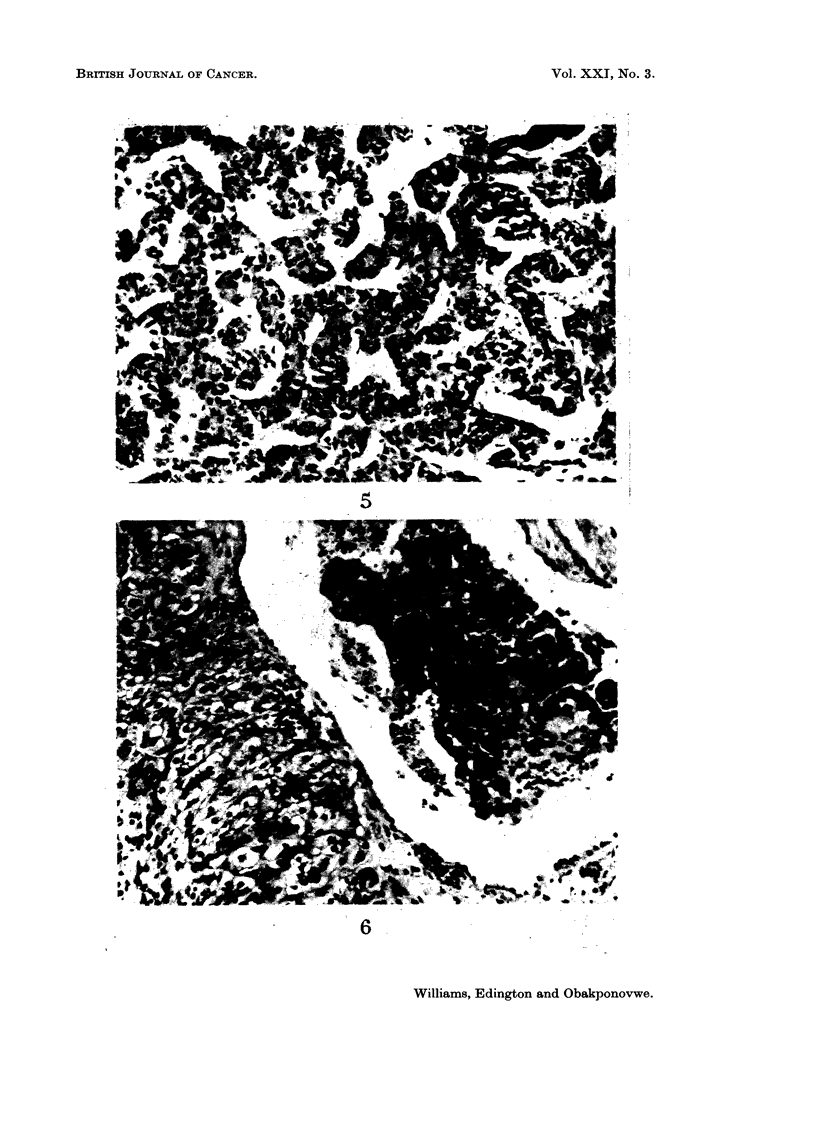

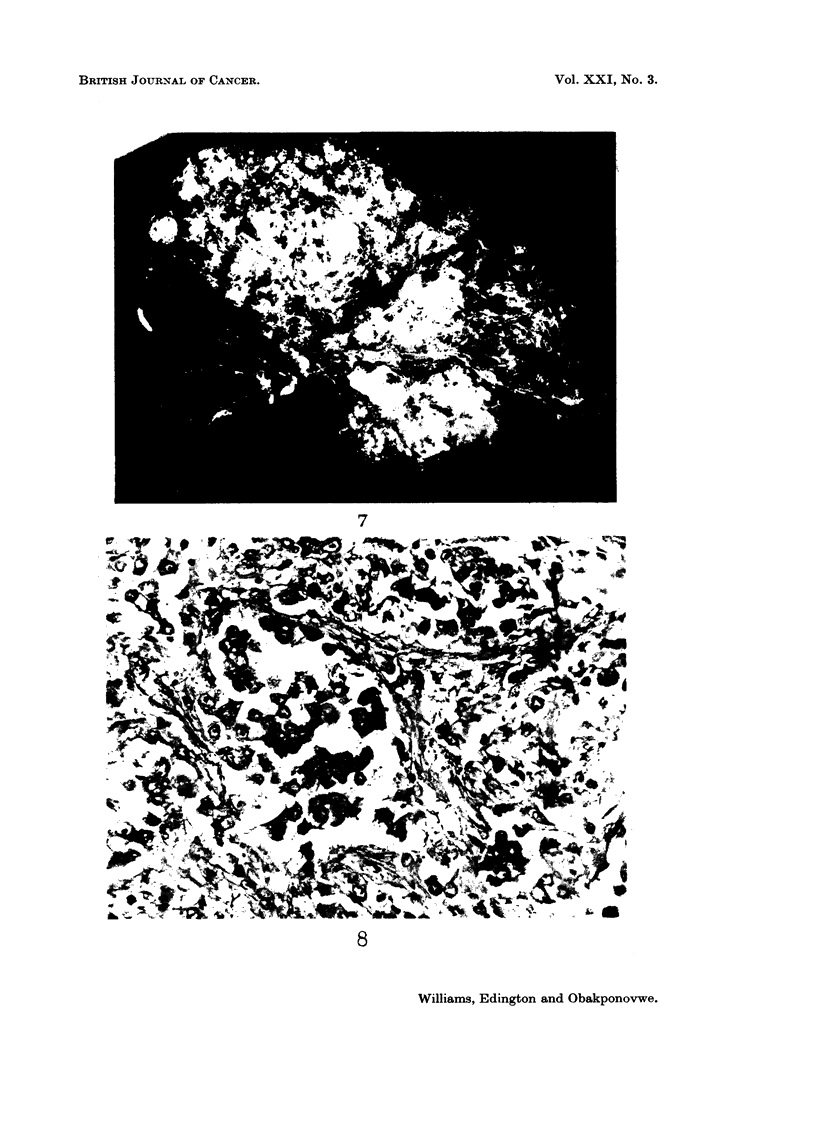

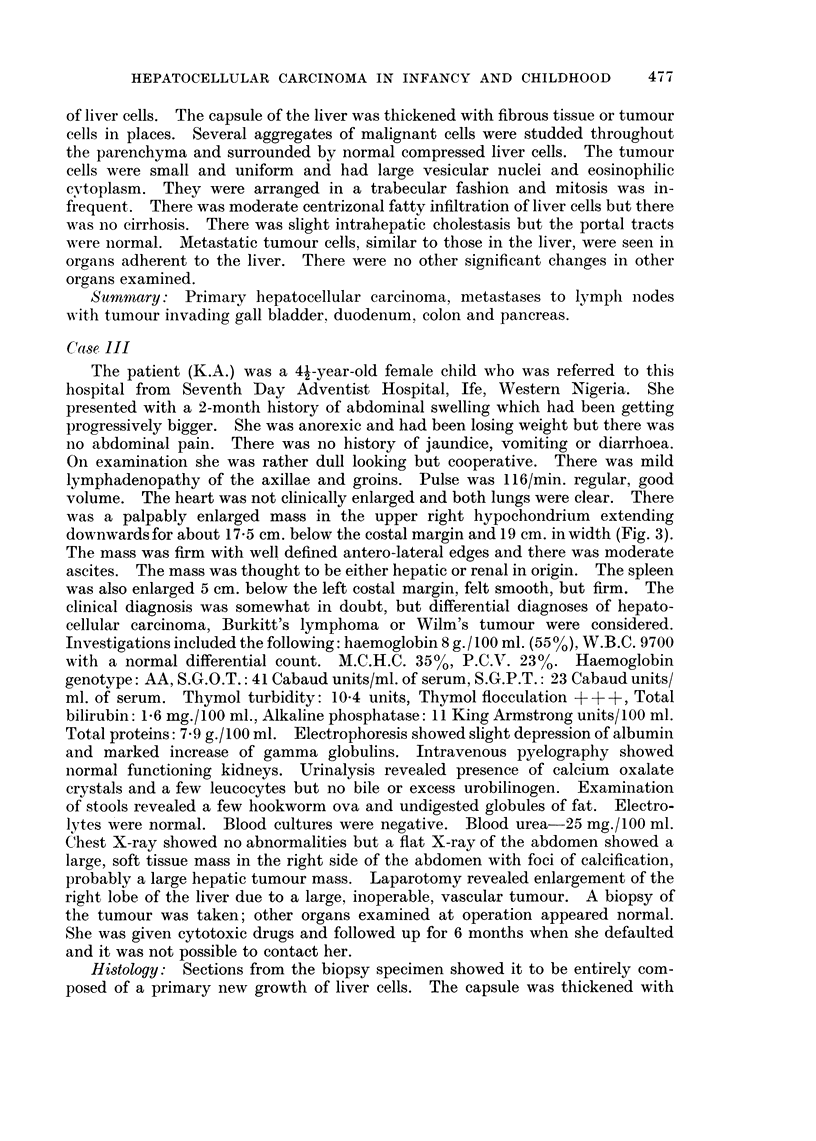

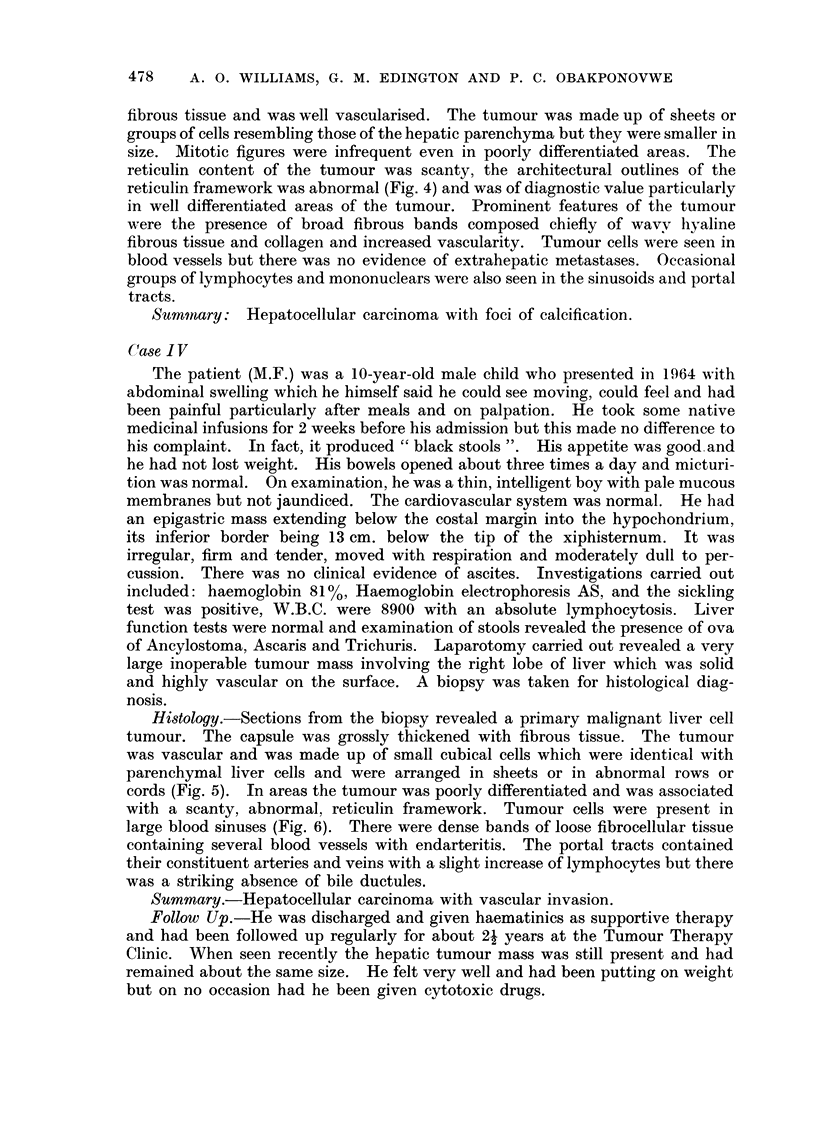

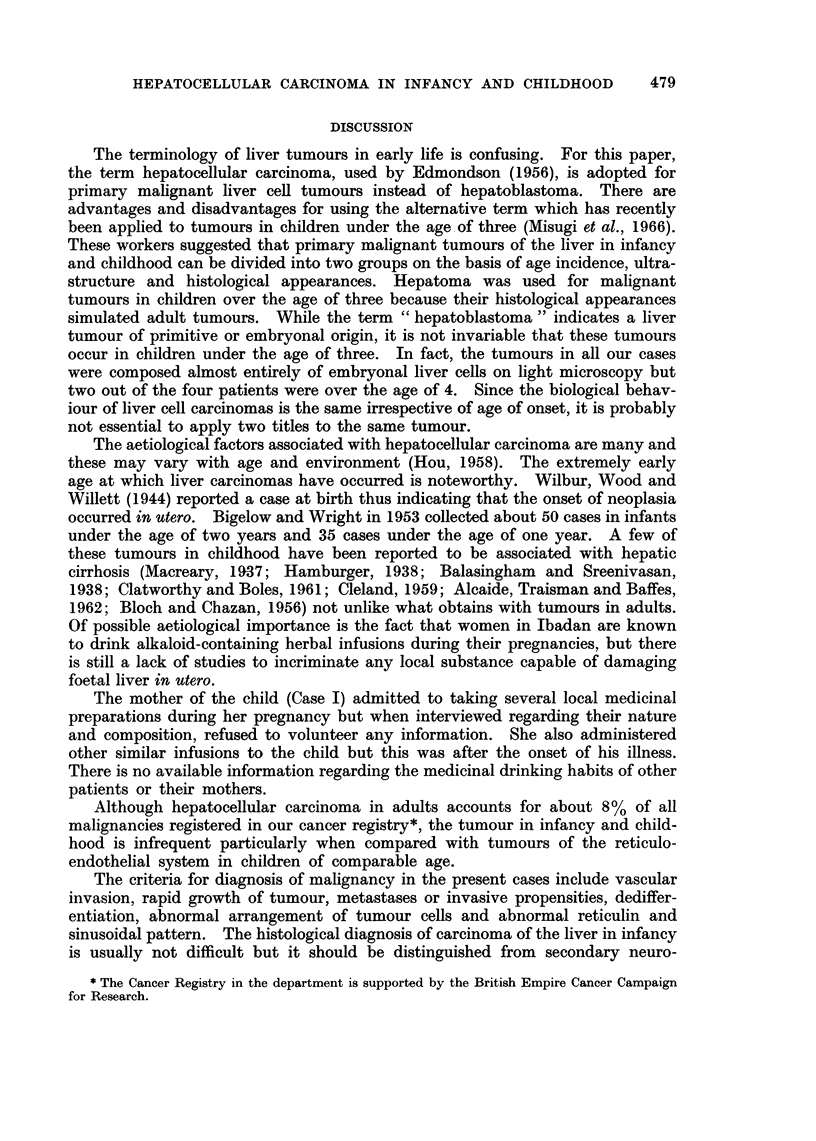

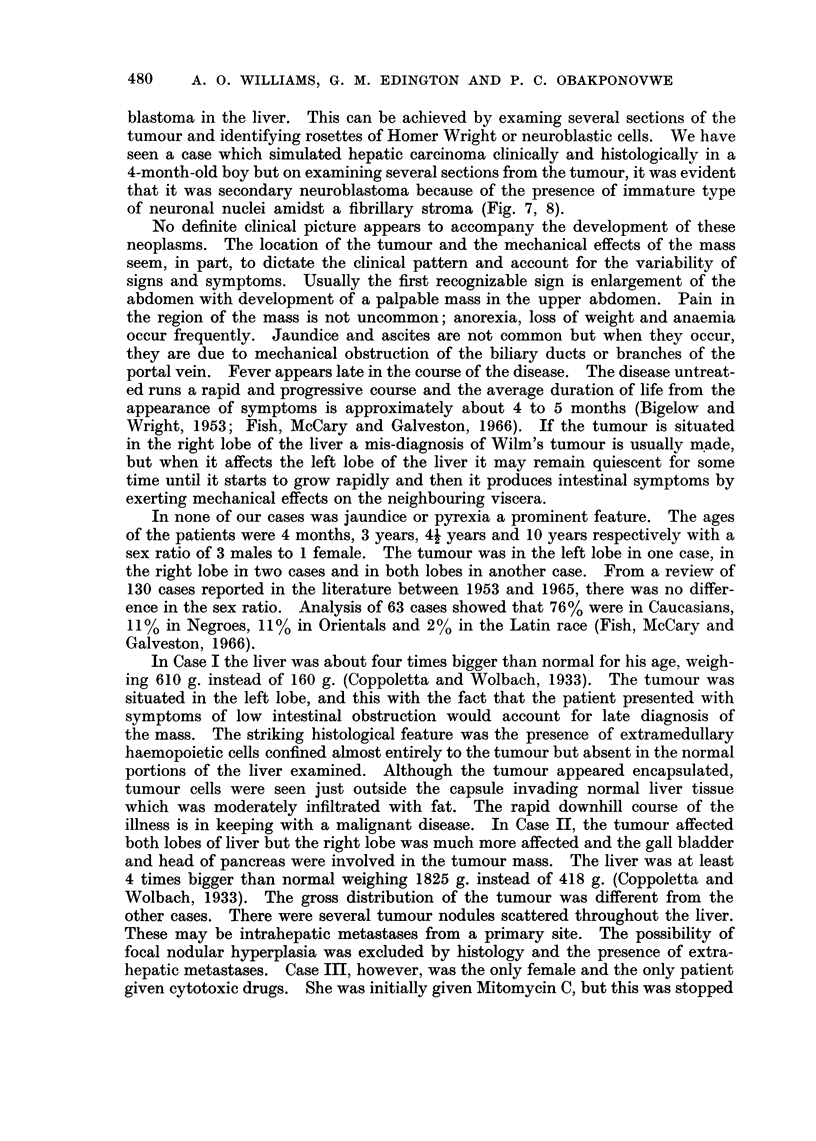

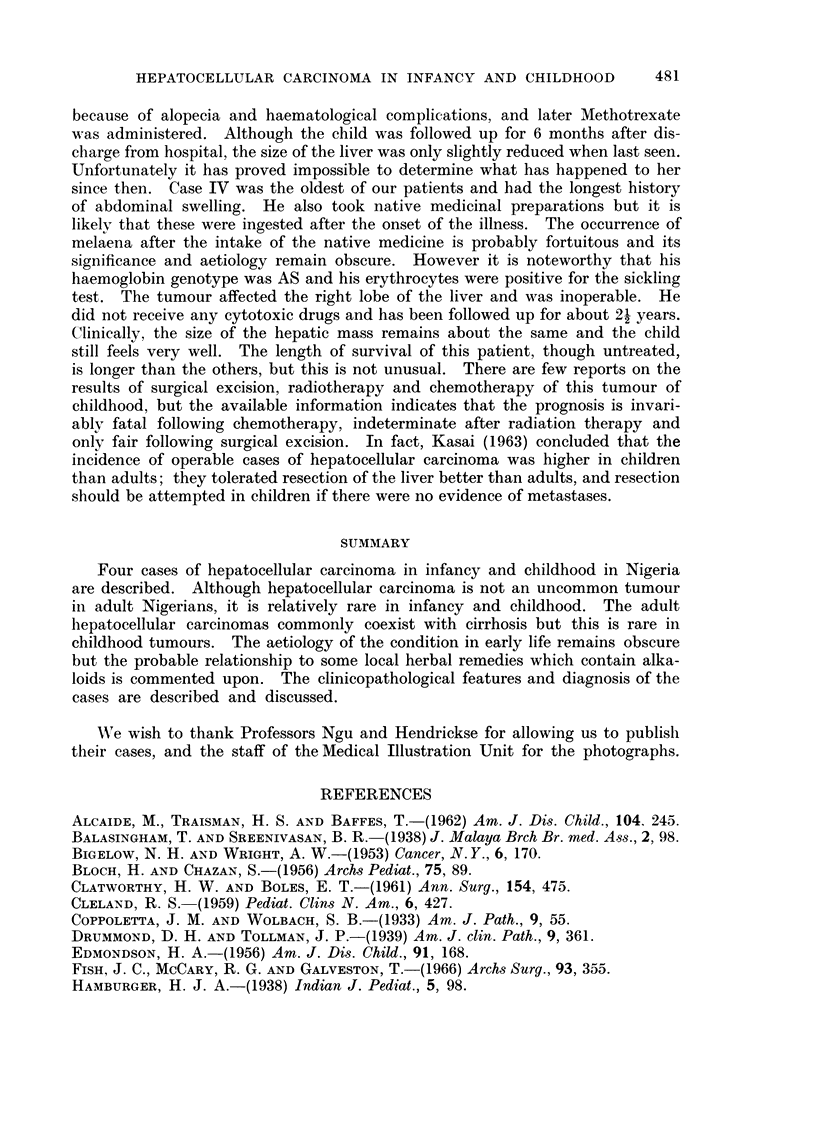

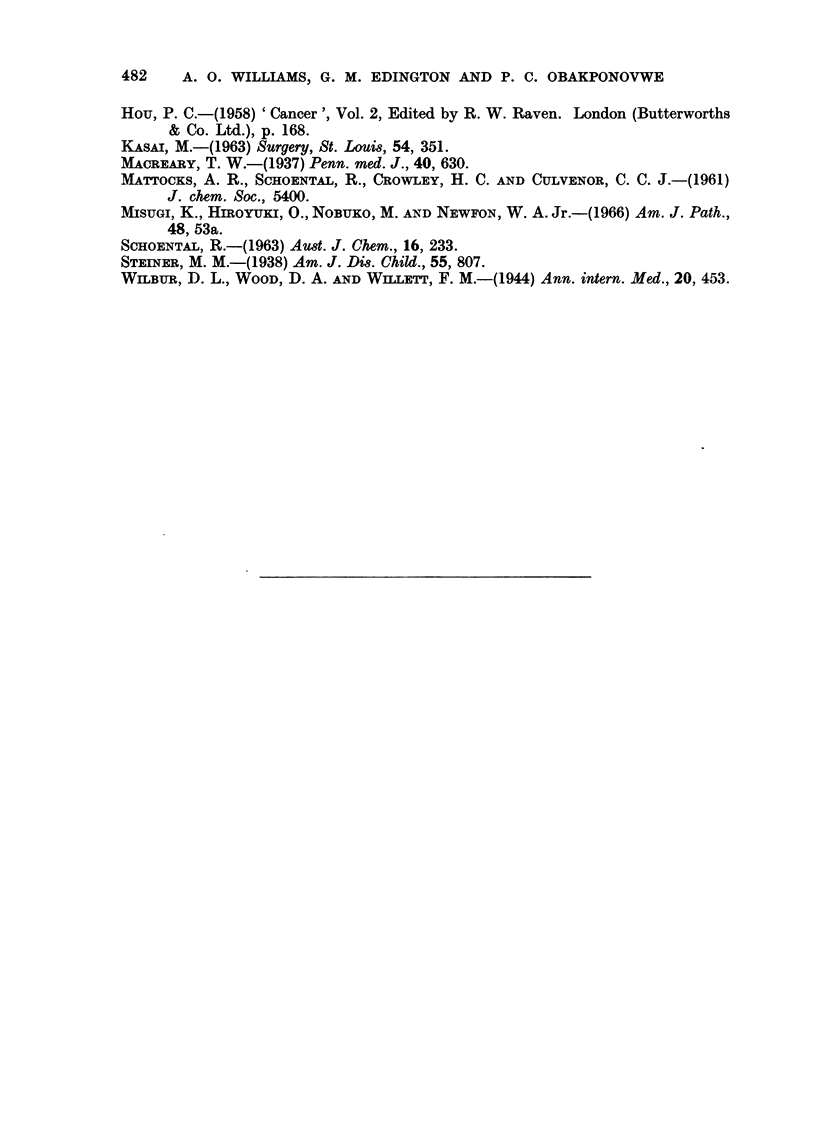


## References

[OCR_00493] ALCALDE V. M., TRAISMAN H. S., BAFFES T. (1962). Primary carcinoma of the liver in infancy and childhood.. Am J Dis Child.

[OCR_00498] CLATWORTHY H. W., BOLES E. T., KOTTMEIER P. K. (1961). Liver tumors in infancy and childhood.. Ann Surg.

[OCR_00507] Fish J. C., McCary R. G. (1966). Primary cancer of the liver in childhood.. Arch Surg.

